# Histone Deacetylase Inhibitors Restore Cancer Cell Sensitivity towards T Lymphocytes Mediated Cytotoxicity in Pancreatic Cancer

**DOI:** 10.3390/cancers14153709

**Published:** 2022-07-29

**Authors:** Chin-King Looi, Li-Lian Gan, Wynne Sim, Ling-Wei Hii, Felicia Fei-Lei Chung, Chee-Onn Leong, Wei-Meng Lim, Chun-Wai Mai

**Affiliations:** 1 School of Postgraduate Studies, International Medical University, Kuala Lumpur 57000, Malaysia; 00000028295@student.imu.edu.my (C.-K.L.); lilian.gan@gmail.com (L.-L.G.); 2 Clinical Research Centre, Hospital Tuanku Ja’afar Seremban, Ministry of Health Malaysia, Seremban 70300, Malaysia; 3 School of Medicine, International Medical University, Kuala Lumpur 57000, Malaysia; wynnesim@yahoo.com; 4 Center for Cancer and Stem Cell Research, Development and Innovation (IRDI), Institute for Research, International Medical University, Kuala Lumpur 57000, Malaysia; lingweihii@gmail.com (L.-W.H.); coleong@agtcgenomics.com (C.-O.L.); limweimeng2014@gmail.com (W.-M.L.); 5 School of Pharmacy, International Medical University, Kuala Lumpur 57000, Malaysia; 6 Department of Medical Sciences, School of Medical and Life Sciences, Sunway University, Subang Jaya 47500, Malaysia; feliciacfl@sunway.edu.my; 7 AGTC Genomics, Kuala Lumpur 57000, Malaysia; 8 School of Pharmacy, Monash University Malaysia, Subang Jaya 47500, Malaysia; 9 State Key Laboratory of Oncogenes and Related Genes, Renji-Med X Clinical Stem Cell Research Center, Ren Ji Hospital, School of Medicine, Shanghai Jiao Tong University, Pudong New District, Shanghai 200127, China

**Keywords:** pancreatic ductal adenocarcinoma, cytotoxic T lymphocytes resistance, histone deactylase inhibitors

## Abstract

**Simple Summary:**

Pancreatic ductal adenocarcinoma (PDAC) is an aggressive pancreatic cancer that is resistant to most treatments due to its tumour microenvironment. In search of an effective therapeutic agents that can overcome the tumour microenvironment, we analysed the PDAC patients genomic profilings and identified that patients with high cytotoxic T lymphocytes (CTL) killing activity were associated with better clinical outcomes. Through genomic and proteomic approaches, we identified potential small molecules that may restore CTL activity in PDAC. We validated the findings using two CTL-resistant PDAC cells and concluded that histone deacetylase inhibitors (givinostat and dacinostat) can reverse CTL sensitivity in CTL-resistant PDAC cells.

**Abstract:**

Despite medical advancements, the prognosis of pancreatic ductal adenocarcinoma (PDAC) has not improved significantly over the past 50 years. By utilising the large-scale genomic datasets available from the Australia Pancreatic Cancer Project (PACA-AU) and The Cancer Genomic Atlas Project (TCGA-PAAD), we studied the immunophenotype of PDAC in silico and identified that tumours with high cytotoxic T lymphocytes (CTL) killing activity were associated with favourable clinical outcomes. Using the STRING protein–protein interaction network analysis, the identified differentially expressed genes with low CTL killing activity were associated with TWIST/IL-6R, HDAC5, and EOMES signalling. Following Connectivity Map analysis, we identified 44 small molecules that could restore CTL sensitivity in the PDAC cells. Further high-throughput chemical library screening identified 133 inhibitors that effectively target both parental and CTL-resistant PDAC cells in vitro. Since CTL-resistant PDAC had a higher expression of histone proteins and its acetylated proteins compared to its parental cells, we further investigated the impact of histone deacetylase inhibitors (HDACi) on CTL-mediated cytotoxicity in PDAC cells in vitro, namely SW1990 and BxPC3. Further analyses revealed that givinostat and dacinostat were the two most potent HDAC inhibitors that restored CTL sensitivity in SW1990 and BxPC3 CTL-resistant cells. Through our in silico and in vitro studies, we demonstrate the novel role of HDAC inhibition in restoring CTL resistance and that combinations of HDACi with CTL may represent a promising therapeutic strategy, warranting its further detailed molecular mechanistic studies and animal studies before embarking on the clinical evaluation of these novel combined PDAC treatments.

## 1. Introduction

Pancreatic ductal adenocarcinoma (PDAC) is a highly lethal malignancy and often called the silent killer due to its lack of early and disease-specific clinical symptoms until the disease is advanced [1]. Global Cancer Statistics 2020 (GLOBOCAN) ranks PDAC the seventh leading cause of cancer death in both males and females [2]. With the estimated five-year survival rate of 10% or less [3], pancreatic cancer is predicted to become the second leading cause of cancer death in USA by 2030, surpassing breast, colorectal, and prostate cancers [4]. Despite advancements in surgical techniques, imaging technology, and treatment strategies, there has not been much improvement in patient outcomes. Other than the delayed diagnosis, which contributes to the worst prognosis in PDAC, accumulating evidence indicates that the evasion of host immune surveillance, particularly the T cell-mediated tumour lysis, is a key determinant of tumour progression [5,6,7]. It has been documented that T cell-induced cell death involves the presentation and recognition of tumour antigens that prime the subsequent antitumour immune responses, known as the cancer-immunity cycle [8]. 

Cytotoxic T lymphocytes (CTL) are the most powerful effectors in eliciting antitumour immune response, among the other immune effector cells such as monocytes and natural killer (NK) cells. Multiple studies have demonstrated that increased CTL infiltration is associated with favourable clinical outcomes [9]. The application of immunotherapy, particularly immune checkpoint blockades (ICB) to overcome T cell dysfunction and boost CTL proliferation to eradicate tumour cells, have generated much excitement in numerous cancer types [10,11]. However, immunotherapy has shown to be less effective for PDAC treatment. In fact, PDAC cells can escape from immune recognition and CTL-mediated cytotoxicity through various tumour intrinsic and extrinsic mechanisms, including the loss of immunogenic tumour antigens, non-immunogenic behaviour of tumours due to their poor antigenicity, and formation of highly immunosuppressive tumour microenvironment (TME) [7,12]. Therefore, there is an urgent need to uncover the in-depth pathophysiology behind pancreatic cancer and, thus, to design effective therapeutic strategies to improve the prognosis of PDAC.

With the advances of high-throughput sequencing technologies, big data is becoming available for clinical research and drug development as never before. Multi-omics data, which consist of genomics, epigenomics, transcriptomics, proteomics, and metabolomic information, can be generated from large sets of samples, allowing a comprehensive understanding of human health and diseases at a deeper level [13]. For instance, The Cancer Genome Atlas (TCGA) database provides clinical and genomic information of 33 different cancer types from more than 10,000 tumour specimens [14]. This comprehensive dataset enables researchers to classify tumours by identifying tumour subtypes to understand molecular changes that occur during carcinogenesis and to explain the clinical phenotype variability [14,15]. Indeed, numerous integrative multi-omics studies performed on PDAC have contributed to the further detailed characterisation of PDAC phenotypes, besides allowing the identification of potential biomarkers for diagnosing, predicting survival outcome, and metastatic potential of patients [13,16,17,18]. Moreover, a recent study using multi-omic data revealed that local TME in PDAC can be classified into two variants: the “deserted TME” and “reactive TME”, which exhibit distinct immune phenotypes, tumour-promoting, and chemoprotective functions that can influence clinical outcomes in PDAC [19]. Unfortunately, the identification of potential targets to reverse immune suppression phenotypes in PDAC [6] are still lacking, and thus, further investigations are warranted.

In the current study, we analysed the immunophenotype of PDAC based on the seven-step cancer-immunity cycle [8] and its correlation with tumour prognosis using publicly available data from two PDAC cohorts: TCGA and the International Cancer Genome Consortium Pancreatic Cancer Australian (ICGC, PACA-AU). PDAC patients were then categorised into two groups based on the CTL activity, which significantly correlated with patient prognosis. Next, we conducted a high-throughput screen for small chemical inhibitors that could overcome CTL resistance in PDAC cells. We discovered that histone deacetylase inhibitors (HDACi) alone were able to eradicate both parental and CTL-resistant PDAC cells. HDACs are known to remove acetyl groups and, in doing so, reverse the chromatin acetylation that may repress the transcription of essential genes such as tumour suppressor genes [6,7,20]. The overexpression of HDACs result in the suppression of tumour suppressor genes, contributing to tumour growth and progression whilst the loss of function of HDAC has also been associated with inducing oncogene expression, leading to cancer development [6,7,20]. In this study, we also observed an increase of histone proteins and acetylated histone proteins among the CTL resistance PDAC, indicating the possible role of HDACi in targeting HDAC to reverse CTL resistance. HDACi are commonly classified by its specific ability to inhibit individual HDACs (namely, pan-HDAC inhibitor or Class I- or Class II-specific inhibitor) or some literature may classify them according to its chemical composition [20]. HDACi are also able to target not only the tumour cell itself but also the tumour microenvironment and immune milieu to achieve an anti-tumour effect, making the use of HDACi a promising strategy to eliminate PDAC and eradicate T cell cytotoxicity resistance in PDAC [7,20]. The combination treatment of sublethal doses of HDACi with CTL were shown to enhance PDAC cells’ sensitivity to CTL-mediated killing, suggesting that HDAC may potentially increase tumour immunogenicity and reverse the immune evasion phenotype of PDAC cells. Thus, our findings offer the rationale for HDACi in overcoming the immune resistance in PDAC and augment host antitumour immune response. 

## 2. Materials and Methods

### 2.1. Characterisation of the CTL Activity in PDAC Patients

The TCGA pancreatic adenocarcinoma study (TCGA-PAAD) RNA-sequencing (RNA-seq) dataset and the associated survival data were obtained from the Broad Institute GDAC Firehose site (https://gdac.broadinstitute.org/, accessed on 26 March 2019), while RNA-seq raw counts and the clinical data of patients with PDAC from PACA-AU were downloaded from the ICGC Data Portal (https://dcc.icgc.org, accessed on 27 March 2019). The count values were normalised and presented in FPKM (Fragments Per Kilobase of transcript per Million mapped reads) format. Rows with duplicate gene IDs, unnamed gene ID, or low read-count (>20% zero read counts) were removed. Low tumour purity in the TCGA-PAAD cohort as described in previous study was also excluded from the analyses [19]. Following pre-processing of the raw datasets, the matrix of gene expression profile was generated and used for the subsequent identification of differentially expressed genes. 

Next, the signature gene set containing reference genes involved in the seven-step cancer-immunity cycle [8] was identified based on the tumour immunophenotype (TIP) website (http://biocc.hrbmu.edu.cn/TIP/download/signature%20annotation.txt, accessed on 26 October 2019) and used as the input to estimate the immune activities of each tumour via the TIP portal (http://biocc.hrbmu.edu.cn/TIP/analysis.jsp, accessed on 26 October 2019). The immune activity score was calculated using single-sample gene set enrichment analysis (ssGSEA) [21,22]. The samples from each step of the cancer-immunity cycle were divided into “high” or “low” immune activity score groups based on the mean scores, where values greater than or equal to the mean scores were considered “high”, while immune activity scores lower than the mean were defined as the “low” category. Kaplan–Meier survival analysis and the log-rank test (MantelCox) were performed to compare survival distribution between “high” versus “low” immune activity groups by using GraphPad Prism software (Version 8.0, GraphPad Software, Inc., San Diego, CA, USA). The overall survival (OS) and progression-free survival (PFS) of the “low” and “high” immune activity groups were demonstrated by hazard ratio (HR) and the corresponding 95% confidence interval (95% CI). A total of 69 patients from PACA-AU were included for both OS and PFS survival analysis. However, a total of 119 patients from TCGA-PAAD were used for OS analysis, while only 89 patients from this cohort was used for PFS analysis. Third patients from TCGA-PAAD were excluded for PFS study because of the missing data in this dataset. Clinical factors such as age at diagnosis and gender were also being analysed against CTL activity. A *p*-value of less than 0.05 was deemed as statistically significant. The same software was also used to generate forest plots to compare the hazard ratio of immune activity score with disease progression.

### 2.2. Identification of Differentially Expressed Genes Associated with the Low CTL Activity in PDAC Patients

Differentially expressed genes (DEGs) between high versus low CTL killing activity tumour groups that were statistically significant (Student’s *t*-test, *p* <0.05) between the TCGA-PAAD and PACA-AU cohorts were defined as common gene sets. The identified DEGs were further analysed to decipher and visualise relevant pathways using the STRING (Search Tool for the Retrieval of Interacting Genes/Proteins) database [23] and IPA (Ingenuity Pathway Analysis, QIAGEN Inc., Redwood City, CA, USA https://www.qiagenbioinformatics.com/products/ingenuity-pathway-analysis, accessed on 27 October 2019). The protein–protein interaction (PPI) network was constructed and visualised in the STRING database [23], whereas IPA was performed to identify the top enriched canonical pathways related to low CTL activity in PDAC patients. The differentially regulated gene signature was used for Connectivity map (CMap) analyses, as described in previous studies [24,25].

### 2.3. Determination of Perturbagens That Target PDAC with Low CTL Killing Activity

The identified differentially regulated genes associated with CTL resistance were queried against the CMap database (https://clue.io/, accessed on 27 October 2019) to identify potential candidate drug classes that target CTL resistance and compared gene expression signatures using pattern matching [25]. Perturbagen classes with a connectivity score (tau, τ) of more than −90 were identified as hits. A τ of 90 indicates that only 10% of perturbagens in the reference database showed stronger connectivity to the query [26].

### 2.4. Cell Lines and Cell Cultures

Human PDAC cell lines, BxPC3 and SW1990, were obtained from the American Type Culture Collection (ATCC, Manassas, VA, USA). All cell lines were cultured in RPMI-1640 medium (Corning Inc., Corning, NY, USA) with 10% foetal bovine serum (Sigma-Aldrich, St. Louis, MO, USA), 100 IU/mL penicillin, and 100 µg/mL streptomycin (Biowest, Nuaillé, France). All PDAC cell lines were maintained in a humidified atmosphere with 5% CO_2_ at 37 °C.

### 2.5. Establishment of PDAC Cells with Acquired Resistance towards CTL-Induced Cytotoxicity

The CTL-resistant PDAC cell lines were established by subjecting the parental PDAC cells to multiple rounds of exposure to antigen-independent activated CTLs at the optimised effector to target cells (E:T) ratio of 8:1 (Supplementary Figure S1a). Briefly, isolated CTLs were derived from healthy human whole blood with a density of 2.5 × 10^6^ cells/vial (AvantiCell AsiaPacific, Kuala Lumpur, Malaysia). The cryopreserved CTLs were then thawed and activated for 72 h in a 6-well plate containing X-VIVO™ 15 medium (Lonza Group, Basel, Switzerland) supplemented with 100 U/mL of recombinant human interleukin-2 (IL-2) (GenScript, Piscataway Township, NJ, USA) and human T-Activator CD3/CD28 Dynabeads^®^ (Thermo Fisher Scientific Inc., Waltham, MA, USA). On Day 3 of T cell activation, CTLs were harvested, and CD3/CD28 Dynabeads^®^ were removed magnetically. Anti-EpCAM/anti-CD3 bi-specific T cell engagers (BiTEs) (Creative-Biolabs, Shirley, NY, USA) was added to the harvested CTLs at a final concentration of 1 μg/mL. The target cell culture medium was removed and replaced with fresh culture medium before the addition of activated CTLs. The CTL-induced tumour lysis was allowed for 24 h at 37 °C in a 5% CO_2_ humidified incubator, following which the culture and dead cells were replaced with fresh complete growth medium. The remaining viable cells were allowed to re-populate. Upon recovery, these cells were subjected to an additional two cycles of CTL exposure. The acquired CTL resistance was validated using morphological study and cell viability assay in 12-well and 384-well plates, respectively (Supplementary Figure S1b). The final surviving cells were selected as the CTL-resistant PDAC cell lines, which were then designated as SW1990 CTLr and BxPC3 CTLr.

### 2.6. High-Throughput Chemical Library Screening

A commercially available chemical library (Selleckchem, Houston, TX, USA) consisting of 1672 diverse bioactive small molecules was used to screen for candidate molecules that may target PDAC with acquired resistance against CTL-induced tumour lysis. Briefly, both parental and CTL-resistant PDAC cells were seeded overnight in 384-well opaque plates (5000 cells/well) and treated with 10 µM of each library compound for 72 h. Cell viability was assessed using CellTiter-Glo^®^ Luminescent Cell Viability Assay (Promega Corporation, Madison, WI, USA), as previously described [27]. The luminescent signal was measured by SpectraMax^®^ M3 Multi-Mode Microplate Reader (Molecular Devices, Sunnyvale, CA, USA). Compounds showing greater than 50% of growth inhibition in the parental and CTL-resistant PDAC cells were considered “hits”. The rank distribution of the collective activities based on the known target(s) of the compounds was examined using the redundant siRNA activity (RSA) statistical analysis method [28,29]. The *p*-values were calculated to indicate statistical significance of hit compounds with the same targets being remarkably distributed toward the top-ranking slots [28,29].

### 2.7. Western Blotting

In order to determine the basal expression of histone proteins and acetylated histone proteins in BxPC3 parental and its CTLr cells, the protein lysates were collected, and Western blotting were conducted as per previous establised protocols [24,25]. Briefly, 6 × 10^5^ parental and CTLr cells were seeded overnight. The cells were then lysed in NP40 cell lysis buffer supplemented with protease and phosphatase inhibitors. The total protein content was analysed using Bio-Rad protein assay (Bio-Rad Laboratories, Hercules, CA, USA) according to the instruction manual. Equal amounts of total protein (50 µg) from each sample were separated by different percentage of sodium dodecyl sulfate (SDS)-polyacrylamide gel, in which was 18% (histone H2A, histone H2B, histone H3, acetyl histone H2A, acetyl histone H2B, acetyl histone H3) and 12% (GAPDH). Proteins were electrophoresed using the Mini-PROTEAN^®^ Tetra Cell (Bio-Rad Laboratories, Hercules, CA, USA) at 200 V for 45 min and transferred onto polyvinylidene difluoride (PVDF) membranes (Merck Millipore, Kenilworth, NJ, USA) with the Trans-Blot® SD Semi-Dry Transfer Cell (Bio-Rad Laboratories, Hercules, CA, USA) at 18 V for 60 min. Membranes were blocked with 5% bovine serum albumin (BSA) solution for 2 h at room temperature with gentle agitation and then incubated overnight with the primary antibody at 4 °C with gentle agitation. Histone H2A (D603A), H2B (D2H6), H3B (D1H2), acetyl H2A, acetyl H2B, and acetyl H3-Lys9 (C5B11) primary antibodies (1:1000) were obtained from Cell Signaling Technology, USA, while GADPH primary antibody (1:1000) was obtained from Santa Cruz, CA, USA. For secondary antibodies, horseradish peroxidase (HRP)-conjugated goat anti-mouse polyclonal IgG (1:5000 dilution, Bio-Rad Laboratories, Hercules, CA, USA) or HRP-conjugated goat anti-rabbit polyclonal IgG (1:25,000 dilution, Bio-Rad Laboratories, Hercules, CA, USA) were used. Detection was performed by chemiluminescence using the Amersham ™ ECL ™ Prime Western Blotting Detection Reagent (GE Healthcare Life Sciences, Buckinghamshire, UK). GAPDH was served as a loading control.

### 2.8. HDAC Inhibitor Efficacy Study

In view of the consistent findings directing to HDAC as a potential target in CTL resistance and a higher expression of histones and its acetylated forms among the CTLr PDAC (Supplementary Figure S2), we further evaluate the effect of commercially available pan-HDAC inhibitors, namely belinostat, dacinostat, givinostat, panobinostat, vorinostat, and trichostatin-A (Selleckchem, Houston, TX, USA) in reversing resistance to CTLs [30,31]. Briefly, both parental and CTL-resistant PDAC cell lines were seeded in the 96-well microplates at a density of 500 cells/well and incubated overnight. Cells were treated with 10 µM of each HDACi and incubated for 24 h. Subsequently, activated CTLs were administered at different E:T ratios (1:1, 2:1, 4:1, 8:1 and 16:1). The CTL-induced tumour lysis of PDAC cell lines with HDACi co-treatment was allowed for 24 h in a humidified incubator at 37 °C in 5% CO_2_, followed by termination via the CellTiter-Glo^®^ cell viability assay (Promega Corporation, Madison, WI, USA), according to manufacturer’s instructions. Luminescence was measured using SpectraMax^®^ M3 Multi-Mode Microplate Reader (Molecular Devices Sunnyvale, San Jose, CA, USA). Since all tested HDAC inhibitors were pan-HDAC inhibitors, we performed target prediction using SwissTargetPrediction (http://www.swisstargetprediction.ch, accessed on 21 March 2021) [32] to identify the other possible targets of HDACi. The common predicted targets among the most potent HDACi (givinostat and dacinostat) were compared with the less potent HDACi (vorinostat and trichostatin A). The relevant pathways were then analysed using STRING database [23] and visualised using Cytoscape (3.9.1).

## 3. Results

### 3.1. High CTL Activity Is Associated with Better Overall Survival and Disease Progression in PDAC Patients

**Figure 1:**
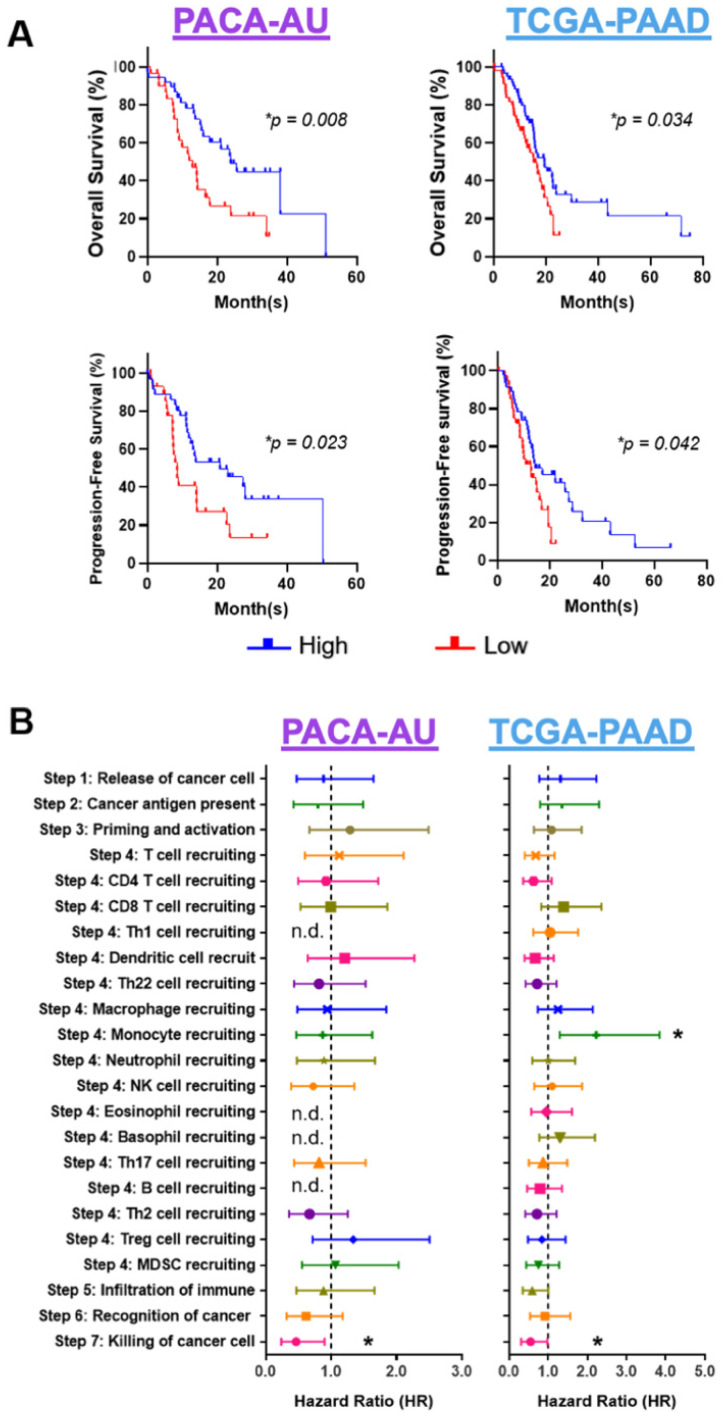
Association of immune activity scores with overall survival. (**A**) High cytotoxic T lymphocytes (CTL) activity (blue line) correlated with better overall survival (OS) and progression-free survival (PFS) in PACA-AU (*n* = 69 for both OS and PFS) and TCGA-PAAD cohorts (*n* = 119 for OS and *n* = 89 for PFS) compared to low CTL activity (red line). (**B**) Forest plots of PFS in relation to immune activity score. Asterisks (*) denote statistical significance at *p* < 0.05; n.d., not determined.

According to the immune activity scores of each step in the cancer-immunity cycle, PDAC patients from both PACA-AU and TCGA-PAAD cohorts were divided into “high” and “low” score groups to evaluate the potential relationship between immunophenotype and prognosis. Kaplan–Meier survival curves showed that an elevated Step 7 immune activity score (killing cancer cells) was significantly associated with better progression free survival (PFS) and overall survival (OS) (*p* < 0.05; Mantel–Cox Log-rank tests) in both the PACA-AU and TCGA-PAAD cohorts (Figure 1A). Based on the PACA-AU cohort, patients with high CTL killing activity demonstrated better OS (*p* = 0.008, *n* = 69) and PFS (*p* = 0.023, *n* = 69) compared to the group with low CTL killing activity. We observed a similar result in the TCGA-PAAD cohort, where groups with high CTL killing activity exhibited better clinical outcomes in both OS (*p* = 0.034, *n* = 119) and PFS (*p* = 0.042, *n* = 89) than the low CTL killing activity group. The immune activity score of 23 sets, seven-step, cancer-immunity cycles for PACA-AU and TCGA-PAAD cohorts was demonstrated by the mean and standard deviation (Figure 1B). High CTL killing activity in both studied cohorts presented a consistent finding of lower HR in developing progressive disease (PACA-AU, HR = 0.46, 95% CI = 0.24–0.90, *p* = 0.023); TCGA-PAAD, HR = 0.55, 95% CI = 0.31–0.98, *p* = 0.042). There were no statistically significant correlations between age at diagnosis or gender with the CTL killing activity (Supplementary Table S1). In summary, the consistent patterns of patients’ outcomes in both cohorts indicate that high CTL killing activity is an independent prognostic marker for better clinical outcomes in PDAC.

### 3.2. Identification of Gene Signature Associated with Low CTL Killing Activity in PDAC Patients

**Figure 2:**
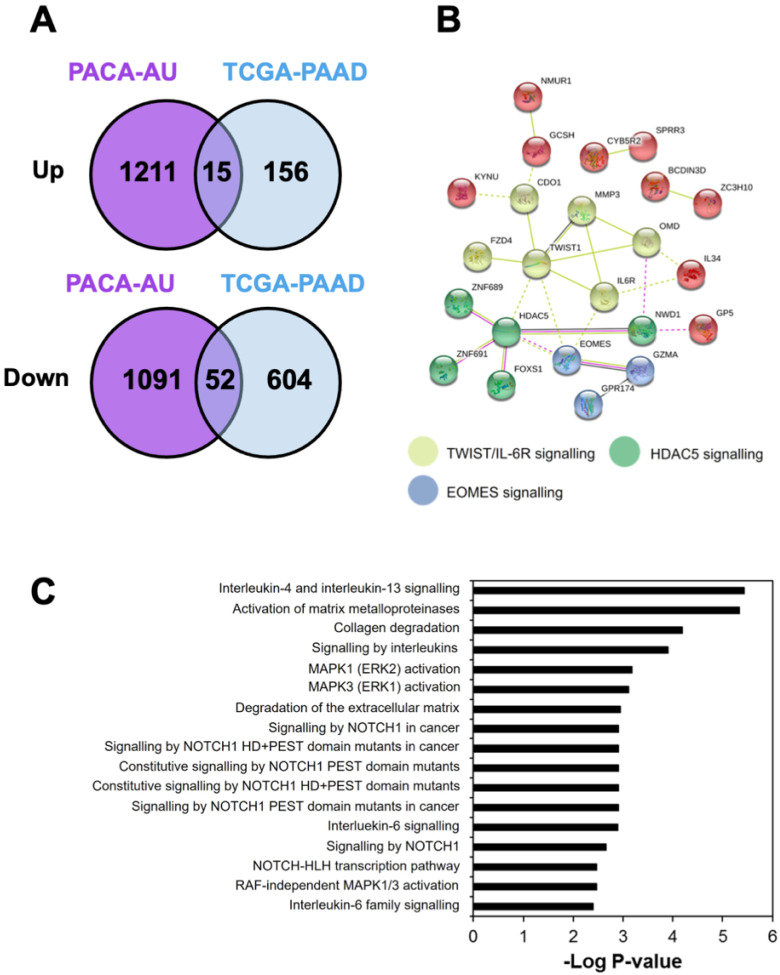
Gene signatures associated with low CTL activities. (**A**) Venn diagram showed the number of up-regulated and downregulated differential expressed genes (DEGs) in PDAC samples with low CTL activity. (**B**) Protein-protein interaction network was constructed using STRING. Three main clus-ters were identified: TWIST/IL-6R, HDAC5, and EOMES signalling as related to low CTL activity in PDAC. (**C**) Bar plots ranking of canonical pathways related to low CTL activity generated from IPA based on the DEGs.

A total of 1226 and 171 upregulated genes (*p* < 0.05, Student’s *t*-test) while 1143 and 656 downregulated genes (*p* < 0.05, Student’s *t*-test) were identified in the PACA-AU and TCGA-PAAD cohorts, respectively. Out of these candidate genes, a total of 15 genes were found to be upregulated while 52 genes were downregulated in tumours with low CTL killing activity in both cohorts (Figure 2A, Supplementary Tables S2 and S3). Three significant clusters that were associated with low CTL killing activity were identified from the STRING (Figure 2B). The yellow cluster was enriched in TWIST/IL-6R signalling; the green cluster was related to histone deacetylase (HDAC)-5 signalling; and the blue cluster was associated with eomesodermin (EOMES) signalling. Further analyses using the IPA and Reactome database revealed that DEGs were significantly enriched in canonical pathways involving: (1) interleukin signalling (especially IL4, IL6, IL13); (2) stromal metabolic activity (metalloproteinases, collagen and extracellular matrix metabolism); (3) mitogen-activated protein kinase (MAPK) activation (MAPK1/ERK2 and MAPK3/ERK1); and (4) NOTCH signalling (NOTCH1) pathways (Figure 2C).

### 3.3. Identification of Perturbagens Targeting PDAC Gene Signature Associated with Low CTL Killing Activity

CMap analysis was performed to identify perturbagens or drugs that could target PDAC with low CTL killing activity. CMap is a reference database consisting of cellular signatures of transcriptional responses of human cells to more than 27,927 small molecules bioactive compounds (termed perturbagens) and more than 1.3 million gene expression signatures [33]. A compounds connectivity score ranging from −100 to +100 is then assigned based on the degree of similarity or dissimilarity, where a negative score indicates dissimilarity between the reference signatures/profiles and query signatures, and a positive connectivity score indicates similarity [24,25,33]. As a result, drugs showing a lower negative connectivity score suggest that the drug yielded signatures that can reverse the query signature and thus were identified as hits [24,25,33]. As shown in Table 1 and Table 2, a total of 44 perturbagens from seven perturbagen classes, including HDACi, phosphoinositide 3-kinase (PI3K) inhibitor, mammalian target of rapamycin (mTOR) inhibitor, insulin-like growth factor (IGF-1) inhibitor, Janus kinase (JAK) inhibitor, DNA dependent protein kinase inhibitor, and Fms-related tyrosine kinase 3 (FLT3) inhibitor, were identified as hits (CMap score ≤ −90). These drugs were found to be related with gene signatures observed in PDAC with low CTL killing activity, suggesting that they possess great potential to reverse CTL resistance in PDAC cells. Notably, HDAC was again being identified as a target responsible for CTL resistance. To confirm the activity of these hits, we performed high-throughput screening assays to identify potential drug candidates that target CTL resistance in PDAC.

### 3.4. Identification of Inhibitors Targeting CTL-Resistant PDAC via High-Throughput Chemical Library Screening

**Figure 3:**
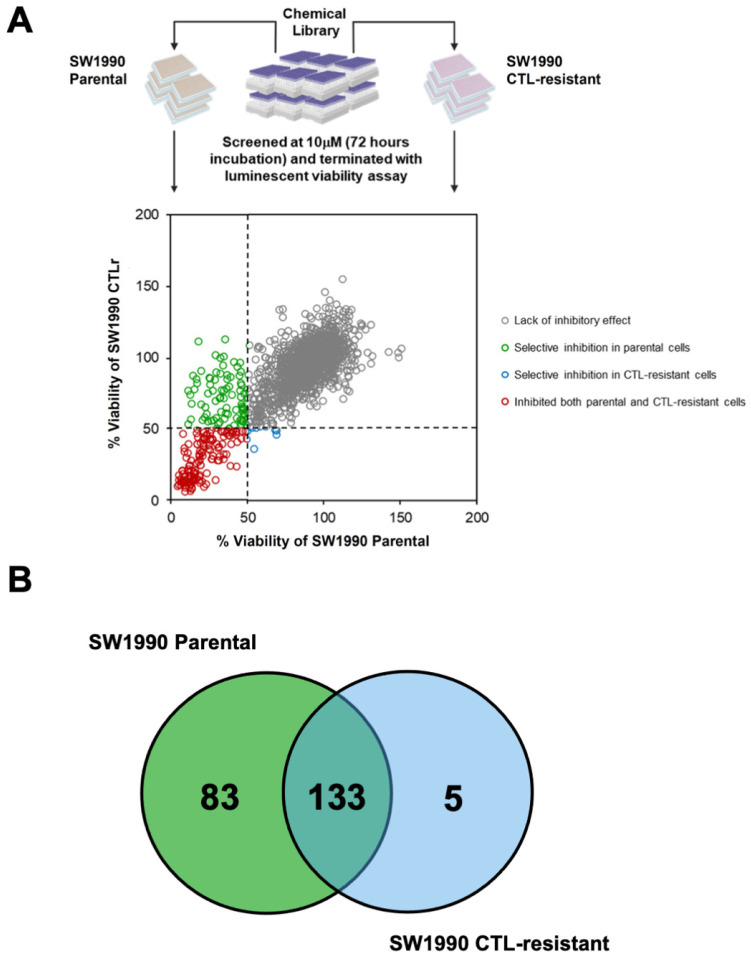
High-throughput phenotypic screen identifies 133 bioactive small molecules targeting both parental and CTL-resistant SW1990 cells. (**A**) A total of 1672 small bioactive molecules were screened in parental and CTL-resistant SW1990 cells at 10 µM for 72 h and terminated with CellTiter-Glo^®^ Luminescent Cell Viability Assay (Promega Corporation, Madison, WI, USA). By combining the screening data from both lines, compounds that exerted inhibitory effects against both parental and CTL-resistant cells were identified. Green circles, molecules targeting parental SW1990 cells only; blue circles, molecules targeting SW1990 CTLr; red circles, molecules targeting both parental and CTL-resistant SW1990 cells; grey circles, molecules lacking anti-proliferative activities. (**B**) Compounds which inhibited both parental and CTL-resistant SW1990 cells (cell inhibitory effect < 50%) were considered “hits”.

A cell-based high-throughput chemical library screen was performed to determine the inhibitory effects of bioactive drug molecules against both parental and CTL-resistant PDAC cells (SW1990 and SW1990 CTLr) (Figure 3A). Out of the 1672 compounds tested, a total of 133 (8.0%) compounds were found to target both parental and CTL-resistant SW1990 cells and were identified as hits (Figure 3B). We identified heat shock protein (HSP), HDAC, proteasome, cyclin-dependent kinase (CDK), and mTOR as the top five targets that, when inhibited, would elicit growth inhibitory effects against both parental and CTL-resistant SW1990 cells (Table 3). Indeed, some of the top-ranking targets, such as HSP [34] and HDAC, have been shown to promote cell survival and tumour progression through the induction of chronic inflammation and suppression of immune cell activity in pancreatic cancer, indicating that these pathways are required for the survival of PDAC cells and evasion of T cell surveillance [6,7]. Notably, HDAC was consistently being identified as the druggable target in CTL resistance PDAC. Therefore, we focused on investigating a panel of HDAC inhibitors to further confirm whether HDACi can reverse CTL resistance in PDAC.

### 3.5. HDAC Inhibitors Restore CTL Sensitivity towards CTL-Resistant PDAC Cells

Epigenetic aberrations have been shown to promote tumour-intrinsic immune evasion in various solid tumours, including pancreatic cancer, and the utility of HDACi has been shown to significantly improve tumour cell sensitivity to gemcitabine, inducing a synergistic antitumour effect on gemcitabine-resistant pancreatic cancer cells. Moreover, HDACi have been demonstrated to upregulate the expression of genes involved in antigen presentation and/or co-stimulatory molecules, which resulted in the enhanced recognition and activation of CTLs [6,7]. 

We further explored the basal expression of the histone proteins (H2A, H2B, and H3) and their acetyl histone proteins in parental and CTLr cells. Interestingly, a higher expression of the histone proteins (H2A, H2B, and H3) and its acetylated forms (H2B and H3) were detected in CTLr cells compared to its parental cells (Supplementary Figure S2). Since histone acetylation should be balanced by both histone acetyl transferase (HAT) and HDAC [6,7], the high expression of both histone (unacetylated or deacetylated) proteins and acetyl histone proteins (Supplementary Figure S2) suggesting a possible disruption of balance between HAT and HDAC activities. Since HDAC was also consistently identified as a druggable target in our in silico analysis (Figure 2B, Table 1 and Table 2) and high-throughput screening (Table 3), we sought to focus the following investigation on HDACi. In this study, we selected six pan-HDAC inhibitors, including belinostat, givinostat, panobinostat, vorinostat, dacinostat, and trichostatin A (TSA), for further testing. Of note, belinostat, panobinostat, and vorinostat have been approved by the United States Food and Drug Administration (FDA) for the treatment of relapsed or refractory peripheral T cell lymphoma (PTCL), multiple myeloma, and cutaneous T cell lymphoma (CTCL), respectively. 

**Figure 4:**
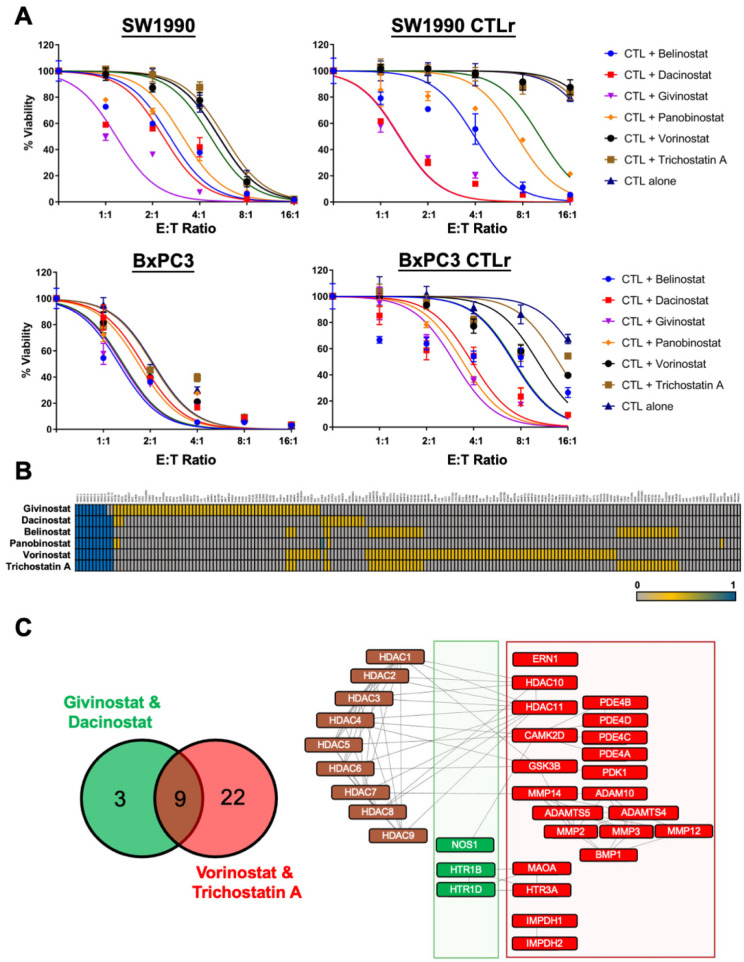
The effects of combinatory pan-HDAC inhibitors and activated CTL in parental and CTL-resistant SW1990 and BxPC3. (**A**) All PDAC cells were pre-treated with HDACi at 10 µM for 24 h, following co-treatment with varying concentrations of CTLs. Points represent mean ± S.D. of at least three replicates. (**B**) Chemical structures of HDACi were imported into SwissTargetPrediction, and a heat map was generated. (**C**) Venn diagram shows the number of common targets in the most potent HDACi (givinostat and dacinostat, green cluster) and the less potent HDACi (vorinostat and trichostatin A, red cluster). Protein–protein interaction network was constructed using STRING.

Briefly, the PDAC cells were pre-treated with the sublethal doses of HDACi alone for 24 h, followed by 24 h co-treatment with varying concentrations of CTL. Notably, SW1990 CTLr and BxPC3 CTLr, having acquired resistance towards CTL-mediated killing, were not statistically significant affected when exposed to CTLs alone, even when exposed to the highest E:T ratio of 16:1, with *p* = 0.284 and 0.142, respectively (Figure 4A). Treatment with CTL alone was shown to exert a dose-dependent cytotoxic effect on the parental SW1990 and BxPC3 cells (Figure 4A). As indicated in our high-throughput screening (Figure 3 and Table 2), HDACi restored CTL sensitivity especially in PDAC CTLr (Figure 4A). Upon HDACi addition, the cell viability of SW1990 CTLr was statistically significant reduced to approximately 5% for belinostat, givinostat, and dacinostat and 25% for panobinostat when co-cultured with CTLs at a E:T ratio of 16:1 (*p* <0.01). These findings highlight the important role of HDACi in restoring CTL sensitivity towards PDAC CTLr. However, no significant restoration of CTL killing effect was observed in the SW1990 CTLr cells treated with vorinostat and TSA. Similarly, a statistically significant cell death of BxPC3 CTLr cells was observed in the presence of HDACi with a 16:1 E:T ratio of CTL, *p* < 0.01 (Figure 4A). At the highest E:T ratio, BxPC3 CTLr cell viability remained at approximately 70% with CTL alone, while the co-treatment with HDACi significantly enhanced the CTL’s sensitivity (*p* < 0.01) (Figure 4A). In particular, givinostat and dacinostat resulted in the complete killing of BxPC3 CTLr cells at an E:T16:1 ratio. On the other hand, belinostat, vorinostat, and TSA showed statistically significant improvement in tumour cell sensitivity towards CTL only at the highest E:T ratio (*p* < 0.01). Both givinostat and dacinostat were more potent than other HDACi, evidenced by a lower ratio of E:T required to induce cytotoxicity in both parental cells and CTL resistance cells. However, vorinostat and TSA were less effective HDACi in enhancing the CTL sensitivity (Figure 4A). SwissTargetPrediction identified that all tested HDACi may target HDAC 1 to HDAC 11, except givinostat, which may not target HDAC 10 and HDAC 11 (Figure 4B, Supplementary Table S4). Givinostat and dacinostat (the more potent HDACi) shared 3 common predicted targets while vorinostat, and TSA (the less potent HDACi) shared 22 common predicted targets, indicating that different HDACi may have slight difference in their ability to restore CTL activity due to its downstream targets (Figure 4B,C, and Supplementary Table S4). Further studies are certainly warranted to explore how’s such targets may affect CTL sensitivity in PDAC CTLr. Taken together, our results indicated that inhibition of HDAC may suppress pancreatic tumour growth and survival by enhancing the functional capacity of CTLs leading to CTL-mediated cell death in PDAC. 

## 4. Discussion

PDAC is recognised as a highly heterogeneous disease consisting of diverse entities with distinct morphologies and clinical behaviours [35]. Despite the presence of many immune cells in the tumour microenvironment (TME), pancreatic cancer patients are frequently associated with immune dysfunction, where the TME is highly immunosuppressive, hence restricting the activation and effector function of T cells, particularly the CTL [6,7]. CTL is the primarily mediators of antitumour immunity by inducing tumour cells lysis [36]. Hiraoka and co-workers highlighted that infiltration of CTL was marked in low-grade premalignant lesions but diminished during the progression of pancreatic intraepithelial neoplasias (PanINs) and intraductal papillary-mucinous neoplasms (IPMNs) [37]. Circulating level of CTL in PDAC patients has been shown to significantly correlate with PDAC progression and patients’ prognosis. Patients with a high density of CTL had significantly prolonged survival compared with those with low CTL infiltration [38,39,40]. Consistent with these studies, our studied cohorts (PACA-AU and TCGA-PAAD) demonstrated that “high” Step 7 (killing of tumour cells), a reflection of CTL activity indicating CTL activity, can be a significant prognostic indicator of OS and PFS in PDAC. 

Nevertheless, it is worth noting that several factors can affect the effector functions of CTL, including: (1) influx of immune suppressive cells; (2) expression of co-inhibitory molecules on tumour cell surface; (3) dysfunctional of T cells; and (4) poor tumour immunogenicity [6,41]. Moreover, previous studies have revealed that tumours continue to proliferate despite the presence of high levels of CTL in the TME of patients [42,43]. As a result, we hypothesised that PDAC tumours with low CTL killing activity may evade immune response and acquire resistance to CTL-mediated cytotoxicity through a tumour cell-intrinsic mechanism. To test this hypothesis, we performed computational genomic analyses to determine molecular mechanisms of resistance and to identify novel immune-modulatory genes that may drive immune evasion in PDAC. Our results indicated that the low CTL activity in PDAC was primarily involved in TWIST/IL-6R, HDAC5, and EOMES signalling. Pathway analyses using IPA further revealed a significant enrichment in the interleukin signalling, stromal activities, MAPK activation, and NOTCH signalling pathways. Our findings are consistent with previous studies reporting the crosstalk between interleukins, MAPK and NOTCH-signalling pathways with CTL resistance [44,45,46]. For instance, inhibition of NOTCH signalling in lung adenocarcinoma [45] and colorectal carcinoma [46] patients augmented the cytotoxicity of tumour-infiltrating CTLs but also promoted perforin and IFN-γ production by activated CTLs to reverse tumour suppression activity. In PDAC, high levels of NOTCH3 expression were correlated with tumour grade, metastasis, tumour-node-metastasis (TNM) stage, and, hence, shortened patients survival time [47]. On the other hand, Notch2 expression was associated with the progression of pancreatic intraepithelial neoplasia (PanIN) in a mouse model [48] by altering the cytokine networks and tumour microenvironments [44]. 

Following the discovery of relevant tumour-intrinsic immune modulators, we next sought to identify potential new treatment options using Connectivity Map (CMap). Through the DEGs related to low CTL activity from both studied cohorts, we identified potential treatment compounds with lower negative connectivity scores (τ > −90) that could potentially reverse CTL resistance. The perturbational class identified in our study, namely PI3K inhibitors [49], mTOR inhibitors [50], IGF-1 inhibitors [51], JAK inhibitors [52], DNA dependent protein kinase inhibitors [53], HDAC inhibitors [54], and FLT3 inhibitors [55], were also reported to mediate immune modulation in various cancers. Further high-throughput chemical libraries screening identified 133 small inhibitors that could target both PDAC parental and CTL-resistant cells. This list includes inhibitors targeting HSP, HDAC, proteasome, CDK, and mTOR signalling. As a growing body of evidence has suggested that pan-HDAC inhibitors are capable of modulating anti-tumour immune responses specifically in PDAC [56,57], a panel of pan-HDAC inhibitors were selected to validate these findings. In the present study, we demonstrated that a lower E:T ratio can achieve the almost complete eradication of PDAC parental cells upon combination treatment with HDACi, suggesting that HDACi may enhance the effector function of CTLs and restore sensitivity towards CTL-mediated killing in cells with acquired resistance. In view of cancer that may have an impact on CTL’s activity [6], it is unclear whether a cancer patient’s CTL may achieve similar synergism with HDACi. A current in vitro study was conducted using healthy donors’ CTLs instead of CTLs from cancer patients. As a result, future study may explore whether the similar synergism can be achieved when the CTLs were isolated from pancreatic cancer patients. 

HDACs are epigenetic enzymes that remove acetyl groups from the lysine residues in histones. Deacetylation of histones promote chromatin condensation, leading to transcriptional repression [58]. To date, a total of 18 HDAC enzymes have been identified in humans and classified into five phylogenetic classes based on homology to yeast deacetylases: class I (HDAC-1, -2, -3, and -8), class IIa (HDAC4, -5, -7, and -9), class IIb (HDAC6 and -10), class III (sirtuins/SIRT1-7), and class IV (HDAC11) [58]. Significant evidence has emerged that the epigenetic dysregulation of immune-related genes/pathways play a crucial role in the development of immune evasion in cancer and resistance to immunotherapies [58]. Moreover, expression of the HDACs is frequently deregulated in various cancers, including breast cancer [59], lung cancer [60], and PDAC [61]. Ideally, histone acetylation is controlled by a balance between histone acetyltransferase (HAT) and HDAC. HAT induces histone acetylation, resulting in an increase of acetyl histone, while HDAC induces deacetylation causing the histone remains not acetylated or deacetylated. In the present study, we observed that the levels of acetyl histone H2B and H3 were significantly higher in CTL-resistant BxPC3 cells compared to parental cells, suggesting that there could be an increase of HAT activity upon acquiring CTL resistance (Supplementary Figure S2). However, we also observed an increase in histone proteins (H2A, H2B and H3) among the CTL-resistant BxPC3 cells, indicating the possibility of an increase in HDAC activity; therefore, the histone remains not acetylated or deacetylated (Supplementary Figure S2). The increase of both histone and acetyl histone proteins clearly indicates a possible disruption of a balance between HAT and HDAC. Aberrant histone acetylation due to the imbalance activity of HATs and HDAC activity within the tumour cells may cause the transcriptional repression of tumour suppressor genes and impair the activation of antitumour immunity, promote immune escape and drug resistance, and ultimately contribute to tumour development and progression [58]. Notably, HDAC activity could be due to non-histone proteins pathways. HDAC can also regulate other transcription factors such as STAT3, TP53, RB, and NF-kB [6,58]. The high acetyl histone levels among CTLr PDAC may also be independent of HAT or HDAC activity. Currently, no study can explain histone acetylation as the result or the cause of an increase in HAT and HDAC activity. The quantification of basal expression of histone and acetyl histone proteins may not truly indicate the complex dynamic interaction among histone acetylation, HAT, and HDAC. To address this limitation, it would be interesting to conduct further investigation to illustrate whether the high histone acetylation in CTLr PDAC is related to an increase of HAT or HDAC. 

HDAC can also influence the antigen presentation via MHC molecules, maturation of dendritic cell function by controlling inflammatory mediator production, cytokine production from activated T cells, homeostasis of Treg cells, and expression of immune checkpoint molecules [58]. Indeed, numerous studies have demonstrated that through the hyperacetylation of histone and non-histone targets, HDACi can restore normal gene expression and protein function, which may enhance tumour immunogenicity and reverse acquired immune resistance [62]. Although there is limited understanding of HDACi’s mechanism of action in pancreatic cancers and its TME, it is believed that the main mechanism of HDACi-effected tumour cell death is the initiation of apoptosis, which may occur through intrinsic (mitochondrial) or extrinsic (death receptor) pathways [20,58]. Intrinsic and extrinsic pathways both eventually cause the activation of caspase and cell demise. Cellular stress stimulates the intrinsic pathway, for example, due to the use of chemotherapy or radiation that causes the disruption of mitochondrial membranes [20]. The extrinsic pathway occurs when ligands, including Fas ligand or TNF, bind to death receptors [20]. HDACi was found to increase the transcription of BAK1 pro-apoptotic genes as well as genes coding for death effector elements, thus enhancing apoptosis [20,58]. Another mechanism of action of HDACi is the blocking of tumour angiogenesis, through blocking tumour angiogenesis by hyperacetylating and degrading hypoxia-inducible factor (HIF-1a) that acts as a proangiogenic transcription factor [20]. As a result, these findings in turn have led to the discovery and development of pan-HDAC inhibitors as potential anticancer therapeutic agents, especially in hematologic malignancies [58]. Vorinostat [63] the first pan-HDAC inhibitor approved by the FDA for the treatment of cutaneous T-cell lymphoma (CTCL). In 2014, belinostat (another pan-HDAC inhibitor) was approved for the treatment against peripheral T-cell lymphoma [64]. The safety and efficacy of HDACi in refractory, advanced, and recurrent solid tumours, including PDAC, are currently undergoing extensive studies [58]. However, as evidenced in multiple failed clinical trials, single agents of HDACi have demonstrated limited clinical utility [58,65]. 

Given the important role of HDAC in modulating antitumour immune response in various cancers [58,66,67,68], the combination of HDACi with other therapeutic agents gains its popularity. In the present study, we demonstrated that belinostat, dacinostat, givinostat, and panobinostat enhanced CTL sensitivity in SW1990 CTLr and BxPC3 CTLr cells (Figure 4A, *p* value < 0.01), suggesting that the antitumour effects of CTL can be enhanced by HDACi. Givinostat and dacinostat (Figure 4A) were shown to be the most effective HDACi in restoring the CTL-mediated killing of PDAC CTLr. Structural targets prediction (Figure 4B,C) illustrated only the most active pan-HDAC inhibitors (givinostat and dacinostat) and may target nitric oxide synthase (NOS), serotonin receptor 1B (HTR1B), and HTR1D. NOS produces nitric oxide to drive T-cell mediated adaptive immunity [69] and to sensitise tumour cells to therapeutic agents [70], indicating the possible mechanisms underlying the ability of HDACi to restore CTL sensitivity in PDAC CTLr. It is well known that HDACi downregulates NOS production [71,72,73,74]. Eli C Lewis’s team demonstrated givinostat reduced nitric oxide production, supporting our findings in which HDACi has a direct impact on NOS [74]. On the other hand, the knockdown of HTR1B and HTR1D could reduce the proliferation and clonogenicity of PDAC [75], indicating another possible mechanism of these HDACi supporting CTL’s cytotoxicity by downregulating HTR1B and HTR1D. Clinically, givinostat is an orally active pan-HDAC inhibitor with anti-inflammatory properties for the treatment of systemic-onset juvenile idiopathic arthritis [76] and polycythemia vera [77]. A phase I clinical trial in healthy subjects revealed that givinostat repeatedly reduces the production of pro-inflammatory cytokines, including tumour necrosis factor-alpha (TNF-α), IL-1β, IL-6, and interferon (IFN)-γ, without affecting anti-inflammatory cytokines production [78]. Givinostat alone [79] or in combination with azacytidine (a DNA methyltransferase inhibitor) [80] was shown to reduce inflammation by suppressing IFN-γ production, resulting in a significant reduction of tumour growth. Nevertheless, there is no specific mechanistic basis of the immune modulatory function of CTL along with givinostat and dacinostat in PDAC, that indicating our study is the first to elucidate its potential in restoring CTL sensitivity in PDAC with acquired CTL resistance. Future study will be warranted to investigate the impact of givinostat or dacinostat on CTL and PDAC, especially on how these HDACi can reverse CTL resistance in PDAC through NOS, HTR1B, and HTR1D pathways in vitro and in vivo. 

Of note, tumour immunogenicity is a crucial step in the initiation of cancer-immunity response. Studies proposed that HDACi may enhance CTL-mediated cytotoxicity and rescue the low response rate to immunotherapy through the transcriptional upregulation of genes involved in antigen-processing and -presenting machinery, as well as immune checkpoint molecules [58,81]. For instance, the combination of dacinostat and pmel-1 adoptive transfer immunotherapy has been shown to significantly improve antitumour activity and prolong host survival through the increased of MHC molecules and antigen presentation by tumour cells [81]. These findings corroborate with our findings, in which CTL and HDACi (including dacinostat) can reverse CTL resistance in PDAC. In melanoma, Woods and co-workers have demonstrated the role of panobinostat in augmenting MHC molecules and differentiation tumour antigen expression, ultimately leading to an increased activation of antigen-specific T cells [82]. Recently, panobinostat induced significant tumour suppression and led to the eradication of most of the tumours in a human pancreatic cancer xenograft model by combining it with dual-specific murine CAR T cell [83]. Recently, a study further corroborates our findings whereby HDAC3 inhibition can enhance CTL’s activity in view of the ability of HDAC3 to inactivate the cytotoxicity and effector differentiation of CTL [84]. These findings indicated the synergistic interaction of pan-HDAC inhibitor and cancer immunotherapy. Likewise, these findings support our data, in which HDACi may have a pivotal role in strengthening the antitumor effect of the immune cells. It would certainly be interesting to conduct a future study on how HDACi affect CTLs as well as other non-cancer cells in the TME using animal study so that we could further optimise the synergism of HDACi with these cells. 

Despite being the less potent pan-HDAC inhibitors in our study, the use of TSA and vorinostat in murine cervical cancer cells and glioma cells, respectively, resulted in an increased expression of MHC class I and multiple components of the antigen presenting machinery in surviving tumour cells, including transporter associated with antigen processing 1 (TAP1), TAP2, low-molecular-mass protein 2 (LMP-2), and LMP-7. Consequently, the treated cells became more susceptible to CTL-mediated tumour lysis [85,86]. Similarly, the pharmacological inhibition of breast and prostate carcinoma cells with either vorinostat or entinostat significantly augmented tumour cell sensitivity to CTL-mediated cell lysis and suppressed tumour proliferation in a dose-dependent manner [87]. Further analysis revealed that vorinostat significantly increased the expression of tumour-associated antigens and components involved in antigen processing, thereby augmenting the tumour recognition and lysis of tumour cells [87]. A probable explanation of its inadequate potency may rely on its lack of activities on NOS, HTR1B, and HTR1D as well as its additional sensitivity towards 22 targets, namely HDAC10, HDAC11, phosphodiesterase (PDE4A, PDE4B, PDE4C, PDE4D), monoamine oxidase (MAOA), inosine-monophosphate dehydrogenase (IMPDH1, IMPDH2), and matrix metalloproteinases (MMP2, MMP3, MMP12, MMP14) (Figure 4B,C). In view of the limited in vitro mechanistic study being covered in this study, further studies are definitely warranted to further investigate the factors affecting the efficacy of HDAC inhibitors in restoring CTL sensitivity in the CTL acquired resistance of PDAC. To translate these findings to the bedside, animal study is in need to elucidate the impact of combined HDACi and CTL in pancreatic TME as well as its desmoplasia. 

## 5. Conclusions

Through an integrated in silico approach, we identified that TWIST/IL-6R, HDAC5, and EOMES signalling were the three main molecular pathways involved in driving CTL resistance in PDAC patients. Through the comprehensive analysis of the genomics data of PDAC, we have identified 44 candidate drugs, including HDACi, that could target CTL resistance in PDAC. Further chemical library screen and analyses also revealed that HDACi were potent inhibitors targeting CTL-resistant PDAC and able to restore the anti-tumour effects of CTL in PDAC CTLr. Our findings indicate that the administration of HDACi might reverse the acquired CTL resistance, implying HDACi as promising drugs for pancreatic tumours that are less or not responsive to T cell-targeted therapy. Nevertheless, more studies are warranted to understand the specific mechanisms of HDACi in enhancing the CTL-mediated killing of PDAC cells for clinical translation. Meanwhile, the potential of HDAC inhibition in preventing the occurrence of CTL resistance in PDAC and further characterisation of immune modulating effect of HDACi on PDAC cells, and its TME, are worth exploring.

## Figures and Tables

**Table 1:** Perturbagens with connectivity score of less than −90.

Rank	Score	Name	Description
1	−98.48	Linsitinib	IGF-1 inhibitor
2	−98.34	AZD-8055	mTOR inhibitor
3	−98.09	Palbociclib	CDK inhibitor
4	−97.89	Apicidin	HDAC inhibitor
5	−97.85	HG-5-113-01	Protein kinase inhibitor
6	−97.71	KU-0063794	mTOR inhibitor
7	−97.43	Selumetinib	MEK inhibitor
8	−97.37	HSP90-inhibitor	HSP inhibitor
9	−97.11	PI-828	PI3K inhibitor
10	−96.93	PI-103	mTOR inhibitor
11	−96.89	ENMD-2076	FLT3 inhibitor
12	−96.44	PIK-90	PI3K inhibitor
13	−96.33	WYE-354	mTOR inhibitor
14	−96.22	Panobinostat	HDAC inhibitor
15	−96.16	Dactolisib	mTOR inhibitor
16	−95.53	Scriptaid	HDAC inhibitor
17	−95.17	ISOX	HDAC inhibitor
18	−95.14	GDC-0941	PI3K inhibitor
19	−94.99	Aminopurvalanol-a	Tyrosine kinase inhibitor
20	−94.94	Lestaurtinib	FLT3 inhibitor
21	−94.73	GSK-1059615	PI3K inhibitor
22	−94.59	Topotecan	Topoisomerase inhibitor
23	−94.36	MK-2206	AKT inhibitor
24	−94.24	Staurosporine	PKC inhibitor
25	−94.15	NCH-51	HDAC inhibitor
26	−94.14	PP-1	SRC inhibitor
27	−93.94	PP-30	RAF inhibitor
28	−93.9	Vorinostat	HDAC inhibitor
29	−93.81	TG-101348	FLT3 inhibitor
30	−93.42	BMS-536924	IGF-1 inhibitor
31	−93.41	Fostamatinib	SYK inhibitor
32	−93.13	OSI-027	mTOR inhibitor
33	−93.05	THM-I-94	HDAC inhibitor
34	−92.53	AKT-inhibitor-1-2	AKT inhibitor
35	−92.39	Camptothecin	Topoisomerase inhibitor
36	−92.19	Temsirolimus	mTOR inhibitor
37	−91.99	Idarubicin	Topoisomerase inhibitor
38	−91.6	VER-155008	HSP inhibitor
39	−91.54	NU-7441	DNA dependent protein kinase inhibitor
40	−90.96	ALW-II-38-3	Ephrin inhibitor
41	−90.77	PHA-793887	CDK inhibitor
42	−90.53	TPCA-1	IKK inhibitor
43	−90.49	KU-0060648	DNA dependent protein kinase inhibitor
44	−90.24	Vemurafenib	RAF inhibitor

**Table 2:** Perturbagen class and hits with connectivity score of less than −90.

Rank	Score	Perturbational Class
1	−98.13	PI3K inhibitor
2	−98.03	mTOR inhibitor
3	−96.04	IGF-1 inhibitor
4	−93.68	JAK inhibitor
5	−91.78	DNA dependent protein kinase inhibitor
6	−91.76	HDAC inhibitor
7	−90.11	FLT3 inhibitor

**Table 3:** Top ten targets of hit compounds identified to inhibit both parental and CTL-resistant PDAC.

Rank	Target	Hits	Log *p*-Value (RSA)	
			Parental SW1990	CTL-Resistant SW1990
1	HSP	10/12	−9.77	−10.74
2	HDAC	13/22	−9.24	−9.44
3	Proteasome	6/9	−6.03	−6.31
4	CDK	9/16	−9.59	−5.44
5	mTOR	6/29	−6.71	−5.10
6	Microtubule associated	1/8	−4.56	−4.96
7	Aurora kinase	7/23	−2.95	−4.50
8	Bcr-Abl	3/12	−3.23	−4.45
9	EGFR	6/31	−6.62	−4.27
10	c-Met	4/18	−2.01	−3.46

## Data Availability

The data presented in this study are available upon reasonable request.

## Supplementary Materials

The following supporting information can be downloaded at: https://www.mdpi.com/article/10.3390/cancers14153709/s1, Figure S1: Optimizing effector:target cell (E:T) ratio for effective CTL-mediated killing of PDAC cells; Figure S2: Expression of histone proteins and their acetylated forms on parental and CTL resistance PDAC; Table S1: Correlation of age and gender on CTL activity using PACA-AU and TCGA-PAAD; Table S2: List of genes that were upregulated in PDAC with low CTL killing activity; Table S3: List of genes that were downregulated in PDAC with low CTL killing activity; Table S4: Predicted targets of HDAC inhibitors.
