# Supplementary material for: Histone Deacetylase Inhibitors Restore Cancer Cell Sensitivity towards T Lymphocytes Mediated Cytotoxicity in Pancreatic Cancer
Source: Cancers (Basel). 2022 Jul 29;14(15):3709. doi: 10.3390/cancers14153709 (PMC9367398; doi:10.3390/cancers14153709)
Supplement: Supplementary file 1 [file cancers-14-03709-s001.zip › Supplementary Figure S2.pdf]

**Figure S2: Expression of histone proteins and their acetylated forms on parental and CTL resistance PDAC**

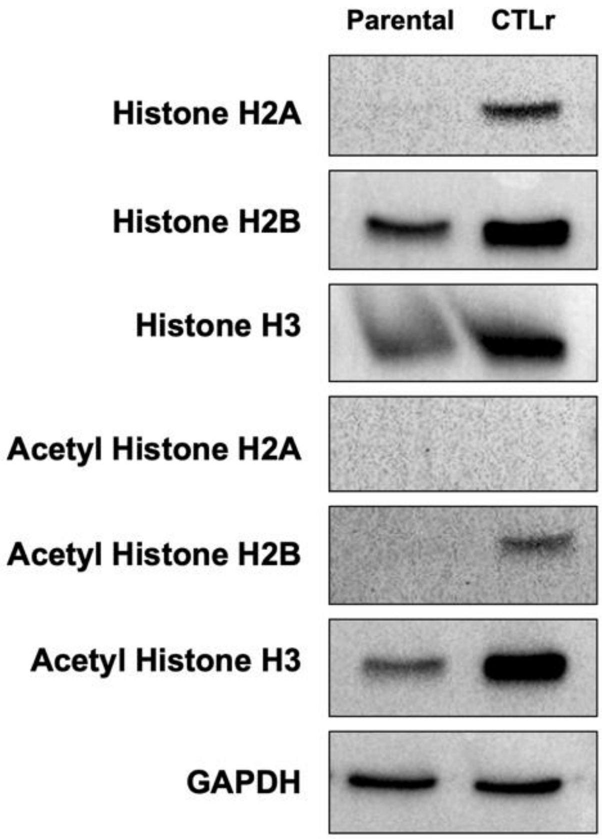


Protein lysates of parental and CTL-resistant (CTLr) cells BxPC3 cells were collected. In order to determine the basal expression of the histone proteins (H2A, H2B, and H3) and their acetylated form, we collected protein lysates of parental and CTLr cells. Interestingly, a higher expression of the histone proteins (H2A, H2B and H3) were detected in CTLr cells. We also observed a significant upregulation of acetylated levels of histone H2B and H3 in CTLr cells as compared to their respective parental cells.
