# Supplementary figures and images for: Histone Deacetylase Inhibitors Restore Cancer Cell Sensitivity towards T Lymphocytes Mediated Cytotoxicity in Pancreatic Cancer
Source: Cancers (Basel). 2022 Jul 29;14(15):3709. doi: 10.3390/cancers14153709 (PMC9367398; doi:10.3390/cancers14153709)

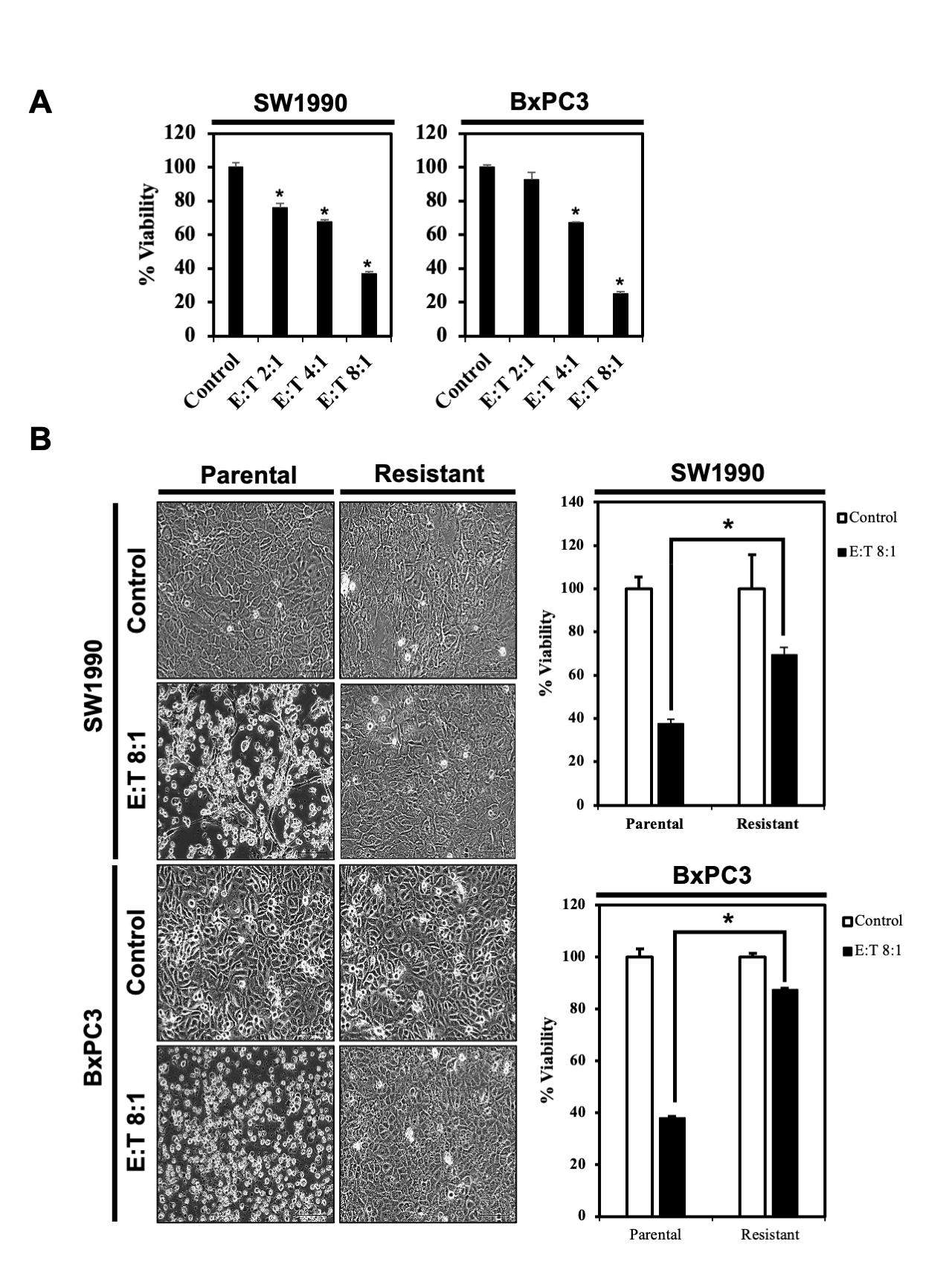

Supplement: Supplementary file 1 [file cancers-14-03709-s001.zip › Supplementary Figure S1.tiff]
